# A commentary on “Application of artificial intelligence lesion labeling system-assisted endoscopic submucosal dissection for the treatment of esophageal lesions in a low-volume center: a prospective cohort study”

**DOI:** 10.1097/JS9.0000000000003077

**Published:** 2025-07-16

**Authors:** Tao He, Lijuan Zuo, Shanming Sun

**Affiliations:** Department of Gastroenterology, Weifang People’s Hospital, Shandong Second Medical University, Weifang, Shandong, China

*Dear Editor*,

We read with great interest the recent article titled “Application of artificial intelligence lesion labeling system-assisted endoscopic submucosal dissection for the treatment of esophageal lesions in a low-volume center: a prospective cohort study”^[1]^ published in the *International Journal of Surgery*. This study aims to confirm that this method is effective in increasing the rate of histological complete resection, particularly for novices at low-volume institutions. The findings demonstrated the potential to increase the complete lateral resection rate of ESD for esophageal lesions in low-volume endoscopy centers, as well as the AI lesion-labeling system’s dependable safety and effectiveness in helping to determine the margin of lesions prior to ESD. Future studies must, however, address a number of important concerns.

First, the study does not include information regarding the size, diversity, and validation of the particular training datasets, although mentioning that the AI system is built on VGG-16 and a multi-task neural network. For example, it does not specify whether the training data includes images from different races, lesion types, or lighting conditions, which could affect the model’s generalization ability. Additionally, key performance parameters, such as the system’s latency in real-time operations and its adaptability to different NBI images, are not clearly defined. The lack of transparency in technical details may hinder other research teams from replicating or optimizing the system.

Second, compared to the traditional group, the AI-assisted group’s ESD operation time was considerably longer (82 minutes vs. 49 minutes, P<0.001). However, the authors attributed this to the operator’s lack of experience and the time required to confirm markers. The study did not further analyze whether the operational complexity of the AI system, such as interface interaction and marker accuracy verification, affected efficiency. Additionally, the study did not explore the learning curve effect—whether the time could be reduced as operators become more familiar with the AI system. This limitation may limit the practical value of AI in clinical settings.

Third, the study lacked long-term follow-up data, like as survival and local recurrence rates for the next 3 to 5 years, and only reported short-term pathological outcomes, such the negative margin rate. The long-term efficacy of esophageal ESD is a critical indicator for evaluating the value of AI systems, particularly in assessing the potential risk of delayed recurrence in low-grade lesions. The authors noted that follow-ups were conducted through outpatient visits and phone calls, but did not provide specific time points or dropout rates, making it difficult to assess the long-term robustness of the conclusions.

This study has demonstrated the auxiliary value of the AI marking system in esophageal endoscopic submucosal dissection (ESD), particularly in low-volume centers, where it can improve the negative margin rate. However, the study has limitations, including non-randomized design, opaque technical details, and a lack of long-term data. Future research should validate the system through randomized controlled trials (RCTs), refine technical documentation, conduct long-term follow-up and cost analysis, and enhance data sharing ethics^[2,3]^. All of this was noted in relation to the TITAN guideline, which calls for transparency in the reporting of artificial intelligence^[4]^.

## Data Availability

No data was used in this Letter to the Editor.
